# Artificial Neural Networks in Lung Cancer Research: A Narrative Review

**DOI:** 10.3390/jcm12030880

**Published:** 2023-01-22

**Authors:** Elena Prisciandaro, Giulia Sedda, Andrea Cara, Cristina Diotti, Lorenzo Spaggiari, Luca Bertolaccini

**Affiliations:** 1 Department of Thoracic Surgery, IEO, European Institute of Oncology IRCCS, 20141 Milan, Italy; 2 Department of Oncology and Hemato-Oncology, University of Milan, 20122 Milan, Italy

**Keywords:** lung cancer, NSCLC, artificial neural network

## Abstract

Background: Artificial neural networks are statistical methods that mimic complex neural connections, simulating the learning dynamics of the human brain. They play a fundamental role in clinical decision-making, although their success depends on good integration with clinical protocols. When applied to lung cancer research, artificial neural networks do not aim to be biologically realistic, but rather to provide efficient models for nonlinear regression or classification. Methods: We conducted a comprehensive search of EMBASE (via Ovid), MEDLINE (via PubMed), Cochrane CENTRAL, and Google Scholar from April 2018 to December 2022, using a combination of keywords and related terms for “artificial neural network”, “lung cancer”, “non-small cell lung cancer”, “diagnosis”, and “treatment”. Results: Artificial neural networks have shown excellent aptitude in learning the relationships between the input/output mapping from a given dataset, without any prior information or assumptions about the statistical distribution of the data. They can simultaneously process numerous variables, managing complexity; hence, they have found broad application in tasks requiring attention. Conclusions: Lung cancer is the most common and lethal form of tumor, with limited diagnostic and treatment methods. The advances in tailored medicine have led to the development of novel tools for diagnosis and treatment. Artificial neural networks can provide valuable support for both basic research and clinical decision-making. Therefore, tight cooperation among surgeons, oncologists, and biostatisticians appears mandatory.

## 1. Introduction

Lung cancer is the most common malignancy and the leading cause of tumor death worldwide [1]. Recent estimates have shown 5-year survival rates of approximately 16% in the United Kingdom [2] and 18% in the United States [3]. In fact, due to the late onset of symptoms, patients are usually diagnosed with lung tumors at an advanced stage, and most of the treatment options turn out to be ineffective [4]. The high prevalence and lethality of lung cancer have led to the development of screening programs for early diagnosis and prognostic stratification systems, intended to improve therapeutic strategies and, consequently, survival outcomes. Computational models have found wide application in this field, as they allow the processing of large amounts of data and provide strong support for clinical decision-making. Artificial neural networks (ANNs) have successfully been used to assess potential prognostic factors and predict survival time and postoperative complications in lung cancer patients [5].

This review aims to provide an updated view of the potential applications of ANNs to the identification of risk factors and the assessment of prognosis and survival in lung cancer patients.

## 2. Methods

We conducted a comprehensive search of EMBASE (via Ovid), MEDLINE (via PubMed), Cochrane CENTRAL, and Google Scholar from April 2018 to December 2022, using a combination of keywords and related terms for “artificial neural network”, “lung cancer”, “non-small cell lung cancer”, “diagnosis”, and “treatment”. The search was restricted to English-language articles only. In addition to the electronic search, a manual examination of meeting abstracts, papers, and reference lists for original and review publications was conducted. Records identified through the designed search strategy were imported into reference management software. In case of duplication, the most recent paper was selected. Two reviewers (E.P. and L.B.) independently screened the titles and abstracts of all of the imported articles. Only papers describing the applications of ANNs to the diagnosis and treatment of lung cancer were included. Some of the excluded papers were retained for discussion. The data extracted for each article included study characteristics, baseline patient characteristics, study period, inclusion criteria, main results, and limitations. The search strategy summary is presented in Table 1.

## 3. Results

### 3.1. Artificial Neural Networks for Dummies

**Figure 1:**
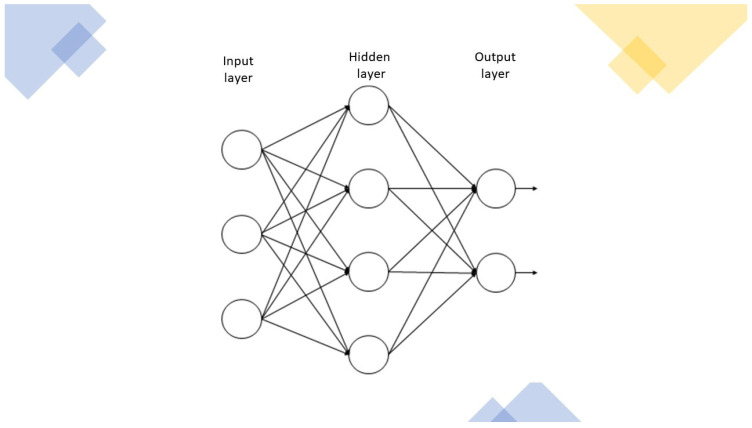
Schematic diagram of an artificial neural network structure with one hidden layer.

**Figure 2:**
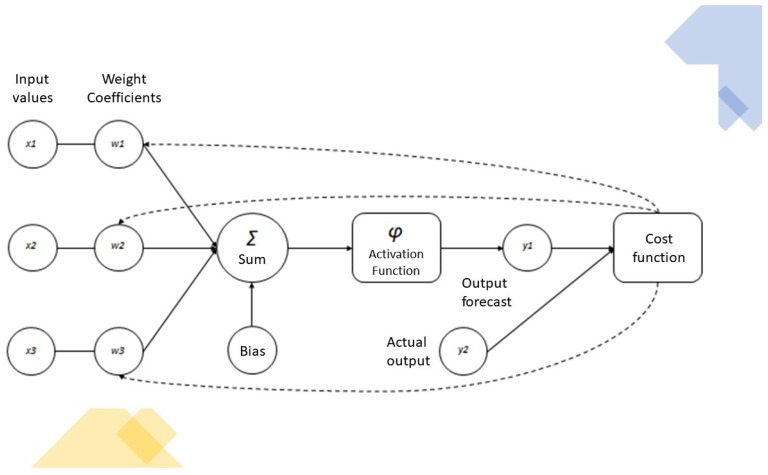
Diagram showing how the neural network processes the input to generate an output signal.

ANNs mimic biological nervous systems, in which neuron nodes are interconnected like a web. Neurons (or nerve cells) are the basic structural and functional unit of the nervous system. They communicate with one another via electrical signals called action potentials. Neurons generate action potentials (outputs) in response to stimuli (inputs). However, not every stimulus is powerful enough to evoke an action potential—only when they exceed a certain threshold (i.e., depolarization of the neuronal membrane) do incoming stimuli elicit an action potential in the receiving (post-synaptic) neuron. Therefore, if the input does not reach the threshold, the output cannot be generated, while once the input rises above the threshold value, an output is always generated. A stimulus that is able to elicit an output signal is called a supraliminal stimulus. The output encoded by a neuron can be considered to be a probabilistic nonlinear function of the input. When the threshold is reached, all-or-nothing electrochemical conduction is started, whereby the neuron can “fire” a signal that travels to other cells. Neuronal responsiveness is not proportional to the intensity of the stimulus but, rather, to the spiking frequency, i.e., the rate at which the neuron fires action potentials. In neural networks, the fundamental computational units are called nodes, organized in layers. Each node receives input signals from nodes of a previous layer of the neural network (or directly from the raw dataset), processes them, and then sends output signals to one or more nodes located in a deeper layer. ANNs have a variable number of layers; a single-layer network is called a simple perceptron network, whereas deep learning networks are more complex neural networks and are composed of several interconnected layers [6]. In fact, between the input and output layers, there may be one or more intermediate layers, called hidden layers, which are responsible for processing the data by applying nonlinear functions. Hidden layers play a key role in enabling an ANN to train on complex tasks and achieve high levels of performance (Figure 1). Because of the intricate organization of ANNs (i.e., high variability of layer numbers and interlayer connections), they are challenging to analyze and are often referred to as “black boxes”. As we have no insight into the functions underlying the learning model, it is not always possible to understand how the ANN generates a specific result. The connection between different nodes is called a synapse. Synapses are extremely dynamic; as they “learn” new input–output maps, they can provide different responses to inputs (synaptic plasticity). We refer to the strength of the connection between two or more nodes as synaptic weight. Synaptic weights are partially responsible for the problematic model interpretation of ANNs, i.e., two ANNs trained with the same dataset and network structure but with different synaptic weights may obtain the same result. In fact, the weight is an essential property of the synapse, as it provides a measure of the effect that the input has on the receiving node and the overall neural network. A node multiplies each input by its synaptic weight, and then it adds up the resulting values to obtain a weighted sum that is then added to (positive or negative) biases. The total is fed to an activation function to produce an output [7]. Finally, the output passes through a cost function, and the results are fed back to the network (Figure 2). This internal feedback enables ANNs to “learn by themselves”—like the human brain, they repeatedly check the differences between the anticipated results and the actual values obtained at the end of the process and can modify their synaptic connections accordingly. Backpropagation is a training method by which synaptic weights are adjusted, based on the error rate of the previous iteration of the input-to-output process [8]. The advantages of ANN models include the following:

1. Rapid recognition of linear patterns, nonlinear patterns with threshold impacts, and categorical, stepwise, and contingency effects.
2. High fault tolerance.
3. Overcoming noisy or incomplete input patterns.
4. Capacity to solve problems whose solution is too complicated or non-algorithmic.
5. The ability to generalize from the training data.
6. The possibility of starting analysis in the absence of hypotheses or predetermined key variables.

Hence, ANNs are particularly suited for tasks requiring focus awareness and can contribute to the implementation of clinical protocols [5].

### 3.2. Artificial Neural Networks in Lung Cancer Research

Lin and colleagues first applied ANNs for lung cancer diagnosis [9] to reduce the false-positive rate in automatic pulmonary nodule detection on X-rays. Since then, ANNs have been increasingly employed in lung cancer research, owing to their unique characteristics. Here, we report some examples of applications of ANNs in lung cancer research.

#### 3.2.1. Risk Factors and Diagnosis

Early detection of lung cancer and identification of at-risk individuals is of utmost importance to prolong survival. To predict and stratify lung cancer risk, Hart et al. [10] developed a multiparameter ANN model with an architecture of 13, 13, 13, and 1 nodes in the input, hidden, and output layers, respectively (i.e., 13-13-13-1), using clinical and demographic information commonly found in electronic medical systems. The validation set had a sensitivity of 75.3% and a specificity of 80.6%.

In the study by Xie et al. [11], an ANN and fivefold cross-validation were used to identify risk factors for lung cancer. The authors selected 15 predictive variables (e.g., radius, sphericity, average intensity) with an accuracy of 83.8%. The number of hidden neurons was not provided; therefore, it was difficult to assess possible sources of overfitting.

Yu and colleagues [12] employed an ANN as a benchmark in the study of a gene expression programming model for the auxiliary diagnosis of small-cell lung cancer. In their analysis, gene expression programming showed higher accuracy than an ANN. However, as in the study of Xie and colleagues [11], the number of hidden neurons was not specified.

Feng and colleagues [13] built four backpropagation ANN models for discriminating lung cancer from benign pulmonary disease and three distinct types of gastric cancer. They combined 6 tumor markers and 19 parameters (layer architecture 25–15–1 or 6–15–1). As regards the differential diagnosis between lung cancer and benign disease, the resulting sensitivity, specificity, and accuracy were 98.3%, 99.5%, and 96.9%, respectively. The ANN proved to be a reliable tool for predicting survival when clinical parameters and genetic polymorphisms were included in the model.

Naresh et al. [14] applied an ANN model and two other classifiers (support-vector machine and k-nearest neighbors) to the computed tomography (CT) detection of early-stage lung cancer. The accuracy of the ANN was 92.7%. However, the performance of this model might have been overestimated, because only a training–test procedure (without validation) was performed.

Uthoff and colleagues [15] also worked with chest CT to explore the advantages of using perinodular characteristics as input variables for machine learning tools trained in discriminating malignant and benign lung nodules. Their findings suggested that such radiological features significantly improved the ANN model’s performance (*p* < 0.01). The authors reported 100% sensitivity and 96% specificity using standardized feature extraction regions from the pulmonary parenchyma.

Zhan et al. [16] developed an intelligent medical system with sensors based on convolutional neural networks to assist in the diagnosis of patients with non-small-cell lung cancer (NSCLC). Word vector matrices were pretrained on medical record text data of patients using a Skip-gram model; then, the deep learning algorithm was employed to extract semantic features. A training algorithm was designed, combining transfer learning and dynamic sampling, and the model was trained on small samples. The authors observed that the diagnostic accuracy of the proposed model increased with the sample data size, approaching the accuracy of the physician. Specifically, with a case sample size of 8000, the accuracy of the system was 84%, while the physician’s accuracy was 86%. Therefore, the authors concluded that when the size of the training case sample was large enough, the system was able to diagnose patients with NSCLC with comparable accuracy to that of clinicians.

Chen and colleagues [17] designed a predictive model to differentiate peripheral small-cell lung cancer (SCLC) from NSCLC based on CT radiomics. They retrospectively collected the clinical records and chest CT scans of patients with pathologically proven and peripherally located SCLC or NSCLC (adenocarcinoma). The scans were semi-automatically segmented by trained radiologists in order to identify the locations of the tumors by indicating the region of interest. Inter- and intra-observer reproducibility and stability for tumor segmentation were assessed and deemed acceptable. A total of 20 radiomic features (out of 1731) were selected to build the ANN model. Finally, a neural network with three hidden layers for nonlinear mapping (architecture 10-7-5) was developed. The input layer consisted of 20 nodes receiving the 20 selected radiomic features, while the output layer consisted of 2 nodes (for the “SCLC” and “NSCLC” decisions). The overall performance of the model was evaluated using the area under the receiver operating characteristic curve, which was approximately 0.93, with a sensitivity of 0.85 and a specificity of 0.85. The authors showed that the reliability of the ANN was improved by employing a combination of radiomic and clinical features rather than individual characteristics. They concluded that the CT radiomic approach might find application as a non-invasive imaging biomarker to differentiate lung cancer.

#### 3.2.2. Postoperative Morbidity and Prognosis

Various machine learning models have been proposed to predict postoperative complication rates and the likely disease course for implementing management and therapeutic strategies. Santos-Garcia et al. [18] designed a group of 100 feedforward ANNs to predict cardiorespiratory morbidity in 515 patients who underwent lung resection for NSCLC. The layered architecture was 13–20–1 or 13–30–1, and the accuracy of the ANN ensemble was 98%, demonstrating that these models provided high performance for morbidity prediction.

In the study by Toney et al. [19], the evaluation of the lymph node size was combined with other previously explored ANN input parameters (i.e., primary tumor maximum standardized uptake value or tumor uptake, tumor size, and lymph nodes’ uptake) to build a backpropagation ANN with an 8-8-4 architecture, which could predict N stage. The accuracy was 99.2%, versus 92.2% for the expert reader (*p* < 0.001).

Chatzimichail and colleagues [20] developed a feedforward ANN with increasing inputs and decreasing hidden neurons to evaluate clinical and molecular prognostic factors by adding a new DNA damage response marker in patients with early-stage lung cancer. The accuracy at 1, 3, and 4 years was 93.3%, 78.7%, and 92%, respectively. The risk of overfitting could not be determined, because the number of hidden layers was not specified.

Chen and colleagues designed [21] a feedforward ANN model with a 9–7 architecture to predict mycotic infections in patients with lung cancer. The accuracy was not determined; however, the authors reported a sensitivity of 58.6% and a specificity of 80.93%. The area under the receiver operating characteristic curve was statistically significantly higher than one of the logistic regression models used for comparison (0.829 ± 0.019 vs. 0.756 ± 0.021; *p* = 0.0041).

Arbour et al. [22] developed a deep learning model to estimate treatment response in patients with advanced-stage NSCLC treated with immunotherapy (checkpoint blockade). They employed the Response Evaluation Criteria in Solid Tumors (RECIST) criteria to assess response to therapy. Trained thoracic radiologists reviewed cross-sectional radiological images to define treatment outcomes according to the standardized RECIST criteria. A deep learning model was then constructed using the clinical text reports describing the RECIST findings of the cross-sectional imaging as inputs. Three different approaches for organizing the input text report data were compared. A fully connected ANN was employed to implement the deep natural language processing model and evaluate each method’s performance. The architecture of the network consisted of an encoding layer, an interaction layer, two fully connected layers, and an output layer. The model was able to correctly estimate complete/partial response, stable disease, and disease progression (according to the RECIST criteria) in 84%, 80%, and 79% of cases, respectively, with a specificity of 96%, 88%, and 93%, respectively. Furthermore, the model accurately determined the occurrence of progression in 85% of cases and the date of progression in 73% of cases (up to 80% prediction of progression occurred within two months). The authors concluded that the proposed model is feasible and accurate and may prove helpful to determine outcomes at scale, thereby allowing studies on large clinical databases.

#### 3.2.3. Survival Assessment

Lung cancer patients’ survival represents an important measure to monitor and evaluate the efficacy of treatments and overall patient care. Hsia and colleagues [23] applied an ANN to assess survival in advanced-stage lung cancer patients and, consequently, to influence treatment strategies. They used a feedforward model with a 25-4-1 architecture; the predicted accuracy was 86.2%.

Poullis et al. [24] investigated the impact of age and body mass index on the 5-year survival of lung cancer patients. They constructed an ANN with a feedforward backpropagation topology and a 12-8-6-2 architecture; however, the accuracy of this model was not reported.

Hou and colleagues [25] constructed a deep learning model with five hidden layers (20-26-32-26-20 architecture) to predict overall survival in patients with pathologically proven NSCLC at all stages. The input layer consisted of eight radiomic features (based on pre-treatment contrast-enhanced chest CT) and five clinical features. The performance of the prediction model was assessed through C-index and time-dependent receiver operating characteristic curves at three timepoints. The authors demonstrated that the model that combined radiomic and clinical features outperformed the models based on clinical or radiomic features alone. Similarly to the study by Chen et al. [17], the prediction efficacy of the combined radiomic + clinical features model was higher than that of the clinical-features-only and radiomic-features-only models.

## 4. Discussion

There are many advantages and disadvantages to using ANNs as classification and regression/prediction tools. They have an excellent aptitude for learning the relationships between the input/output mapping from a given dataset without any prior information or assumptions about the statistical distribution of the data. Learning from datasets without prior knowledge makes ANNs suitable for prediction tasks in clinical situations. Additionally, ANNs are intrinsically nonlinear and, therefore, are useful for accurately modeling sophisticated data patterns. ANNs usually require longer training times than logistic regression models and are also more prone to overfitting due to their broader range of functions [26]. They use a dynamic approach to risk analysis. Despite their limited ability to manage missing values, ANN models can simultaneously process numerous variables, managing complexity (even when processing small and imbalanced datasets) and, thus, avoiding the “dimensionality problem” [5,7].

Moreover, they can consider outliers and nonlinear interactions among variables. As mentioned above, the most common bias of the studies was overfitting—the ANN models performed well on training sets (low error rate) and poorly on the testing data (high error rate). This usually occurs when the model suffers from high variance, resulting in the “noise” (i.e., irrelevant inputs) of the training data being memorized by the ANN. An ANN that is “too well trained” is incapable of generalizing from training data to test data because it is unable to discriminate the “noise” from the relevant inputs.

Radiomics-based ANNs have recently emerged as a promising approach to the diagnosis, prognosis, and survival prediction of lung cancer patients. Selected radiomic features are extracted from conventional radiological images (usually chest CT scans) and then fed as inputs to a deep learning model in order to obtain an efficient, reliable, and non-invasive tool to support patient management. Several potential clinical applications have been described for this novel combined approach; however, its impact on real-world lung cancer management is yet to be confirmed.

## 5. Conclusions

ANNs’ inference has applications in tasks that require focused attention. They are increasingly employed in clinical decision support. However, their success depends crucially on having large amounts of data as inputs and improved integration with research protocols, together with an awareness of the need to combine different paradigms in order to produce the most straightforward and most transparent overall reasoning structure, and the will to evaluate this in a natural clinical environment. This brief overview shows that the studies reporting on the use of ANNs in lung cancer research are often susceptible to bias due to their low accuracy, with a detrimental impact on the reliability of the reported results. ANN models combining radiomic and clinical features have shown promising results, proving that strict cooperation between physicians and biostatisticians is crucial to develop patient-tailored management strategies for NSCLC.

## Figures and Tables

**Table 1:** Search strategy summary of the present narrative review.

Items	Specification
Date of search	31/07/2022
Databases and other sources searched	EMBASE (via Ovid), MEDLINE (via PubMed), Cochrane CENTRAL, and Google Scholar
Search terms used	Combination of keywords and related terms for “artificial neural network”, “lung cancer”, “non-small cell lung cancer”, “diagnosis”, and “treatment”
Timeframe	01/04/2018–31/12/2022
Inclusion and exclusion criteria	(1) English language (2) Papers describing the applications of artificial neural networks to the diagnosis and treatment of lung cancer
Selection process	(1) Records identified through the designed search strategy were imported into reference management software. (2) In case of duplication, the most recent paper was selected. (3) Two reviewers (E.P. and L.B.) independently screened the titles and abstracts of all of the imported articles.

## Data Availability

No new data were created or analyzed in this study. Data sharing is not applicable to this article.
